# *Journal of Experimental & Clinical Cancer Research* reviewer acknowledgement 2013

**DOI:** 10.1186/1756-9966-33-17

**Published:** 2014-02-10

**Authors:** Mauro Castelli

**Affiliations:** 1 Regina Elena National Cancer Institute, Rome, Italy

## Abstract

**Contributing reviewers:**

The editor of *Journal of Experimental & Clinical Cancer Research* would like to thank all our reviewers who have contributed to the journal in Volume 32 (2013).

## 

Michael Abend

Germany

Aaron Adamson

United States of America

Khalil Ahmed

United States of America

Yoshimitsu Akiyama

Japan

Eiman Aleem

Sweden

Cinzia Antognelli

Italy

Vincenzo Arena

Italy

Leon Bach

Australia

Jean-Paul Bahary

Canada

Xuhui Bao

United States of America

Carlton Barnett

United States of America

Luisa Begnozzi

Italy

Sonja Berndt

United States of America

Giovanni Blandino

Italy

Gianluca Bossi

Italy

Christian Bronner

France

Rosaria Bufalino

Italy

Dengfeng Cao

China

Ning Cao

United States of America

Michele Caraglia

Italy

Gaetano Caramori

Italy

Violeta Cardenal

Spain

Alice Carrier

France

Paola Cassoni

Italy

Chiara Castelli

Italy

Pradeep Chaluvally Raghavan

United States of America

Mario Chammas, Jr.

Brazil

Chawnshang Chang

United States of America

Tammy Chang

United States of America

Hui-Ju Ch'ang

Taiwan

Gang Chen

United States of America

Lin-Feng Chen

United States of America

Shih-Shun Chen

Taiwan

Shuda Chen

China

Wantao Chen

China

Liang Cheng

United States of America

Yung Hyun Choi

Korea, South

David Chung

Australia

Mara Cirone

Italy

Martin Clynes

Ireland

Flaminia Coluzzi

Italy

Kim Creek

United States of America

Ziyou Cui

United States of America

Maria Czyzyk-Krzeska

United States of America

Bingbing Dai

United States of America

Alan Dal Pra

Switzerland

Sidsel Damkjær

Denmark

Trina Das

United States of America

Paolo De Fabritiis

Italy

Marina De Resende

Brazil

Kathleen Decker

Canada

Domenico Delia

Italy

Christèle Desbois-Mouthon

France

Benjamin Descours

France

Xiangwu Ding

China

Ruslan Dmitriev

Russian Federation

Hongdo Do

Australia

Chiara Dobrinja

Italy

Jessica Donington

United States of America

Harry Drabkin

United States of America

Jinwei Du

United States of America

Xiao-Li Du

United States of America

Ewald Dumont

Netherlands

Konstantinos Economopoulos

United States of America

Julien Edeline

France

Susumu Eguchi

Japan

Iorio Eugenio Luigi

Italy

Alessandra Fabi

Italy

Xiaolong Fan

China

Huaqiang Fang

United States of America

Francesca Fanini

Italy

Emanuela Felley-Bosco

Switzerland

Gen-Sheng Feng

United States of America

Zizhen Feng

United States of America

Avelino Fraga

Portugal

Jean-Noel Freund

France

Doriana Fruci

Italy

Jianfei Fu

China

Sidney Fu

United States of America

Amy Fulton

United States of America

Nozomu Fuse

Japan

Feng Gao

United States of America

Lina Gao

United States of America

Anna Gasperi-Campani

Italy

Giovanni Gaudino

United States of America

Jing-Shu Geng

China

Antonio Giordano

Italy

Carlo Giordano

Italy

David Gisselsson

Sweden

Munirathinam Gnanasekar

United States of America

Maria Gonzalez Cao

Spain

Philip Gruppuso

United States of America

Anna Gruszka

Poland

Jian Guan

United States of America

Sezgin Günes

Turkey

Jiangfeng Guo

China

Junming Guo

China

Kun Guo

China

Linlang Guo

China

Qifeng Guo

China

Sonal Gupta

United States of America

Jeung-Whan Han

Korea, South

Jianxun Han

Canada

David Hangauer

United States of America

Swei Sunny Hann

China

Huifang Hao

Japan

Noboru Hara

Japan

Bass Hassan

United Kingdom

Etsuro Hatano

Japan

Biao He

United States of America

Yoshihiko Hirohashi

Japan

Gerald Hoefler

Austria

Robert Hoffman

United States of America

Jeff Holly

United Kingdom

Michael Horsman

Denmark

Yingwei Hu

China

Wei-Chien Huang

Taiwan

Xiaojun Huang

China

Yan Huang

United States of America

Fazlul Huq

Australia

Daisuke Iizuka

Japan

Shiro Itagaki

Japan

Chunyan Ji

China

Xinguo Jiang

United States of America

Renjie Jin

United States of America

Chunsheng Kang

China

Shailender Kanwar

United States of America

Sid Kar

United States of America

W. Martin Kast

United States of America

Nancy Kemeny

United States of America

Dong-Wan Kim

Korea, South

Jee Hyun Kim

Korea, South

Dominique Koensgen

Germany

Lingling Kong

China

Manousos Konstadoulakis

Greece

Kaoru Kusama

Japan

Jandee Lee

Korea, South

Sang Joon Lee

Korea, South

Wei Lei

United States of America

Carlo Leonetti

Italy

Karen Leroy

France

Chung-Pin Li

Taiwan

Ji-Liang Li

United Kingdom

Kai Li

China

Min Li

United States of America

Qin Li

United States of America

Rui Li

China

Xiaoli Li

United States of America

Xiuling Li

United States of America

Yu-Chih Liang

Taiwan

Zhiyong Liang

China

Rongxia Liao

China

Weng Lihong

China

Jen-Kou Lin

Taiwan

Yiwei Lin

United States of America

Hubert Lincet

France

Hui Ling

United States of America

Tian Liu

United States of America

Xi Liu

United States of America

Yan Liu

United States of America

Yinkun Liu

China

Yong Liu

United States of America

Zhihua Liu

China

Wen Lian Lo

Taiwan

Pei-Jung Lu

Taiwan

Yong Lu

United States of America

Robert Lucito

United States of America

Paolo Macchiarini

Sweden

Peter Malfertheiner

Germany

Malgorzata Malodobra-Mazur

Poland

Richard Marais

United Kingdom

Jodi Maranchie

United States of America

Ornella Marelli

Italy

Kazumasa Matsumoto

Japan

Fekicity Eb May

United Kingdom

Soeren Torge Mees

Germany

Erhong Meng

United States of America

Michele Menotta

Italy

Leonardo A. Meza-Zepeda

Norway

Paolo Miccoli

Italy

Martin Michaelis

United Kingdom

Shuji Mikami

Japan

Ruben Molina

Spain

Richard Moore

United States of America

Fabiola Moretti

Italy

Marcella Mottolese

Italy

Susan Moug

United Kingdom

Ken-Ichi Mukaisho

Japan

Shigeki Nagamachi

Japan

Binoj Nair

United States of America

Tohru Nakanishi

Japan

Joo-Hyun Nam

Korea, South

Patrick Nasarre

United States of America

Reiki Nishimura

Japan

Shyam Nyati

United States of America

Elena Odintsova

United Kingdom

Sae-Ock Oh

Korea, South

Masayuki Ohtsuka

Japan

Masaaki Oka

Japan

Hirotaka Osada

Japan

Marco Paggi

Italy

Claudia Palena

United States of America

Jianji Pan

China

Lingya Pan

China

Jae Myung Park

Korea, South

Heba Pasha

Egypt

Bo Peng

China

Cheng Peng

United States of America

Marzia Pennati

Italy

Lars J Petersen

Denmark

Cathie Pfleger

United States of America

Ada Piepoli

Italy

Alla Piirsoo

Estonia

Nicola Pimpinelli

Italy

Carme Piñol

Spain

Robert Pirker

Austria

Elena Piskounova

United States of America

George Plataniotis

United Kingdom

Marta Pokrywczynska

Poland

Zhi Rong Qian

Japan

Guixing Qiu

China

Xiaofei Qiu

China

Osvaldo Rampado

Italy

Carlo Reale

Italy

Luigi Ricciardiello

Italy

Mario Roselli

Italy

Antonio Russo

Italy

Kristina Rysevaite

Lithuania

Erica Salvati

Italy

Rosario Sanguedolce

Italy

Daniele Santini

Italy

Anna Sapino

Italy

Ingo Schmidt-Wolf

Germany

Wolfgang Schulz

Germany

Ali Shamseddine

Lebanon

Bal Krishan Sharma

India

Hiroaki Shiba

Japan

Yusuke Shiozawa

United States of America

Wu Shixiu

China

Anuraag Shrivastav

Canada

Zheng Shui-Er

China

Bhuminder Singh

United States of America

Preet Paul Singh

United States of America

Doris Siwak

United States of America

Maja Smerdel

Denmark

Silvia Soddu

Italy

Hai Song

United States of America

Enrico Spugnini

Italy

Edmond Sterpin

Belgium

Carol Stocking

Germany

Subbaya Subramanian

United States of America

Yuan Sun

United States of America

Deepa Taggarshe

United Kingdom

Kohji Takara

Japan

Wayne Tam

United States of America

Manish Tandon

United States of America

Keiji Tanimoto

Japan

Jianguo Tao

United States of America

Rong-Hua Tao

United States of America

Lucio Tentori

Italy

Xing-Song Tian

China

Xinxia Tian

China

Danai Tiwawech

Thailand

Marcos Tobias-Machado

Brazil

Maria Lauda Tomasi

Italy

Gian Paolo Tonini

Italy

Nobuyuki Toshikuni

Japan

Giancarlo Troncone

Italy

Shaw-Jenq Tsai

Taiwan

Shuiping Tu

China

Shahab Uddin

Saudi Arabia

Maciej Ugorski

Poland

Katsuhiro Uzawa

Japan

Antonis Valachis

Greece

Kenneth Van Golen

United States of America

Laura Vargas-Roig

Argentina

Aldo Venuti

Italy

Giovanni Vitale

Italy

Demitrios Vynios

Greece

Christine Walsh

United States of America

Shaogui Wan

United States of America

Congjun Wang

China

Lei Wang

United States of America

Linda Wang

Singapore

Ping Wang

United States of America

Qiuyan Wang

United States of America

Xiaoming Wang

China

Yadong Wang

China

Yan Wang

United States of America

Yingqun Wang

United States of America

Yucai Wang

United States of America

Naoki Watanabe

Japan

Miranda Ween

Australia

Haiming Wei

China

Jun Wei

United States of America

James Wells

Australia

Victor Chun-Lam Wong

United States of America

Sopit Wongkham

Thailand

Jonathan Wright

United States of America

Shaofang Wu

United States of America

Zhengrong Wu

China

Harry Xia

United States of America

Qinghua Xia

China

Junjie Xiao

China

Zhi-Qiang Xiao

China

Zuoxiang Xiao

United States of America

Ke Xu

China

Li Xu

United States of America

Qilin Xu

United States of America

Xiaochun Xu

United States of America

Naoko Yamada

Japan

Libo Yan

United States of America

Eric Yang

United States of America

Gong Yang

China

Leixiang Yang

United States of America

Yibin Yang

United States of America

Yinmo Yang

China

Zhengduo Yang

United States of America

Jun Yao

China

Jun Yasuda

Japan

Huquan Yin

United States of America

Qi Yin

United States of America

Hiroaki Yokomori

Japan

Mee Sup Yoon

United States of America

Kouichi Yoshinari

Japan

Tomokazu Yoshizaki

Japan

Guang-Yan Yu

China

Dong Yuan

United States of America

Zhong Yun

United States of America

Peng Zaimei

China

Kostas Zarogoulidis

Greece

Maria Chiara Zatelli

Italy

Abdel-Rahman Zekri

Egypt

Haibo Zhang

United States of America

Hongyu Zhang

China

Jiangwei Zhang

United States of America

Mingzhi Zhang

China

Qunzhou Zhang

United States of America

Shiwu Zhang

China

Yanling Zhang

United States of America

You-Qing Zhang

United States of America

Zhenwei Zhang

United States of America

Yuhai Zhao

United States of America

Lei Zheng

United States of America

Xiuyi Zhi

China

Bing Zhou

United States of America

Jianwei Zhou

China

Zhongren Zhou

United States of America

Xiaofeng Zhu

China

Xiaolin Zhu

United States of America

Christoph Zielinski

Austria

Wainer Zoli

Italy

Yang Zong

United States of America

Ashraf Zytoon

Egypt

